# Successful Treatment of Persistent and Relapsing COVID-19 with Ensitrelvir in a Patient with Obinutuzumab-Induced Long-Term B-Cell Depletion: A Case Report

**DOI:** 10.3390/reports9010089

**Published:** 2026-03-18

**Authors:** Yoshitaka Haino, Tsuneaki Kenzaka, Tomohiro Hayashi, Kimikazu Yakushijin

**Affiliations:** 1 Department of Internal Medicine, Hyogo Prefectural Tamba Medical Center, 2002-7 Iso, Hikami-cho, Tamba 669-3495, Hyogo, Japan; haino-knz@umin.ac.jp (Y.H.); tomohiro884@hotmail.com (T.H.); 2 Division of Community Medicine and Career Development, Kobe University Graduate School of Medicine, 2-1-5 Arata-cho, Hyogo-ku, Kobe 652-0032, Hyogo, Japan; 3 Division of Medical Oncology/Hematology, Department of Medicine, Kobe University Hospital and Graduate School of Medicine, Kobe 650-0017, Hyogo, Japan; kyakushi@med.kobe-u.ac.jp

**Keywords:** 3CL pro, ensitrelvir, immunocompromised, molnupiravir, persistent COVID-19, refractory COVID-19, remdesivir, case report

## Abstract

**Background and Clinical Significance**: Ensitrelvir is an oral inhibitor of the severe acute respiratory syndrome coronavirus 2 (SARS-CoV-2) main protease (3CL pro). Compared with remdesivir and molnupiravir, ensitrelvir achieves higher rates of SARS-CoV-2 antigen clearance and a more favorable viral shedding profile. **Case Presentation**: A 67-year-old Japanese man with follicular lymphoma had received obinutuzumab plus bendamustine, followed by obinutuzumab maintenance therapy. Hypogammaglobulinemia and profound B-cell depletion persisted for more than 1 year after the final maintenance dose. Three months prior to the current admission, the patient developed coronavirus disease 2019 (COVID-19) and was treated with a 10-day course of remdesivir and dexamethasone. The patient subsequently presented with recurrent COVID-19 pneumonia. Treatment with remdesivir and dexamethasone did not result in clinical improvement, and the SARS-CoV-2 antigen level increased despite adjunctive intravenous immunoglobulin. After ensitrelvir was added to remdesivir, the SARS-CoV-2 antigen levels declined rapidly, and clinical parameters, including fever, inflammatory markers (C-reactive protein), and oxygenation, improved promptly, allowing for discharge. **Conclusions**: Ensitrelvir may be an effective therapeutic option for the treatment of persistent or refractory COVID-19 in immunocompromised patients. Clinicians should recognize that patients treated with obinutuzumab may remain immunosuppressed for several years after therapy.

## 1. Introduction and Clinical Significance

Coronavirus disease 2019 (COVID-19) spread rapidly worldwide, and in November 2021, the severe acute respiratory syndrome coronavirus 2 (SARS-CoV-2) Omicron variant was designated as a variant of concern [1]. Several antiviral agents for SARS-CoV-2 are currently available, including remdesivir [2], molnupiravir [3], and ritonavir-boosted nirmatrelvir [4]. Ensitrelvir is an antiviral agent that inhibits the SARS-CoV-2 main protease (3CL pro) [5]. Ensitrelvir administration shortens the time to resolution of fever, fatigue, and respiratory symptoms by 24 h and reduces the time to SARS-CoV-2 antigen negativity [6]. However, current Japanese guidelines [7] do not recommend ensitrelvir as a first-line therapy for patients with risk factors for severe disease, and reports on its use for protracted or refractory COVID-19 in immunocompromised patients remain limited.

COVID-19 in immunosuppressed patients following B-cell depletion therapy is associated with delayed viral clearance, prolonged symptoms, and a high risk of relapse [8]. Anti-CD20 antibodies such as rituximab and obinutuzumab rapidly induce B-cell depletion, and their immunologic effects persist for extended periods [8]. In immunocompromised patients, insufficient humoral immune responses to SARS-CoV-2 can lead to persistent viral infection, severe disease, and viral evolution within the host, posing significant public health and healthcare–economic challenges.

Here, we describe a patient with follicular lymphoma who underwent R-CHOP therapy followed by obinutuzumab plus bendamustine therapy and continued to exhibit hypogammaglobulinemia and profound B-cell depletion for more than 1 year after the final administration. The patient’s initial COVID-19 episode improved with remdesivir, but incomplete viral eradication in the setting of immunodeficiency likely contributed to disease recurrence. After the second COVID-19 episode, the addition of ensitrelvir to the re-administered remdesivir resulted in a rapid decline in SARS-CoV-2 antigen levels and prompt clinical improvement.

## 2. Case Presentation

We present the case of a 67-year-old Japanese man with a history of follicular lymphoma. His chief complaints were dyspnea and a productive cough. Eight years earlier, the patient had received rituximab, cyclophosphamide, doxorubicin, vincristine, and prednisolone (R-CHOP) therapy for Grade 2 Stage IV follicular lymphoma. Four years earlier, he initiated obinutuzumab plus bendamustine (GB) therapy, followed by obinutuzumab maintenance therapy. Treatment continued until 17 months before the current presentation, during which he remained in complete remission.

Three months before the current presentation, the patient developed COVID-19 pneumonia and was treated with a 10-day course of remdesivir and dexamethasone (6 mg/day). The treatment resulted in clinical improvement. Subsequently, the patient developed organizing pneumonia and was treated with prednisolone. Prednisolone was initiated at 30 mg/day and tapered to 15 mg/day over a period of 3 months. Eight days before the current admission, he developed resting dyspnea and a productive cough and visited a local physician. Elevated C-reactive protein (CRP) levels prompted hospital referral.

The patient’s comorbidities included type 2 diabetes mellitus, hypertension, hypopituitarism with adrenal insufficiency, bronchial asthma, and psychosomatic disorders. He was fully independent in his daily living activities. He had received four doses of the COVID-19 vaccine, with the last dose administered 18 months previously. The patient’s regular medications at presentation included prednisolone (15 mg/day), linagliptin (5 mg/day), candesartan/amlodipine combination tablets (8 mg/2.5 mg), bisoprolol (2.5 mg), montelukast (10 mg/day), clotiazepam (5 mg/day), and folic acid (5 mg/day).

### 2.1. Investigation

On presentation, the patient was alert (Glasgow Coma Scale score: E4V5M6). His vital signs were as follows: blood pressure 121/72 mmHg, pulse 85 beats per minute, temperature 36.3 °C, respiratory rate 16 breaths per minute, and SpO_2_ 94% on room air.

Physical examination revealed fine inspiratory crackles in the left lung and regular heart sounds without murmurs. The abdomen was mildly distended and soft, with slight tenderness in the right upper quadrant but no rebound tenderness.

Laboratory findings (Table 1) included white blood cell count, 1520/μL; neutrophils, 89.5%; lymphocytes, 7.2%; total protein, 5.7 g/dL; albumin, 3.1 g/dL; hemoglobin A1c, 9.9%; CRP, 7.98 mg/dL; immunoglobulin (Ig) G, 589 mg/dL; IgA, 70 mg/dL; IgM, 7 mg/dL; and SARS-CoV-2 antigen, 103 pg/mL. Blood cultures (two sets) were negative, and sputum cultures showed no significant pathogens.

**Figure 1:**
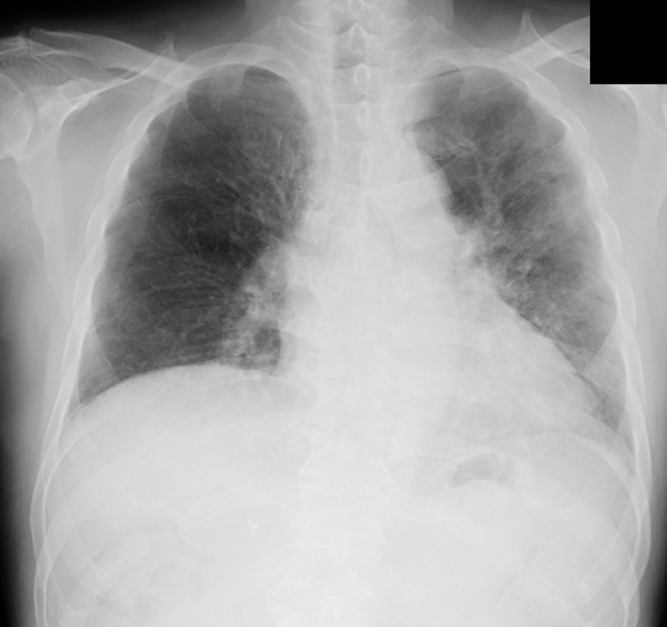
Chest X-ray on admission. Chest radiography revealing a cardiothoracic ratio of 55%, sharp right and dull left costophrenic angles, and diffuse ground-glass opacities in the right lung field.

**Figure 2:**
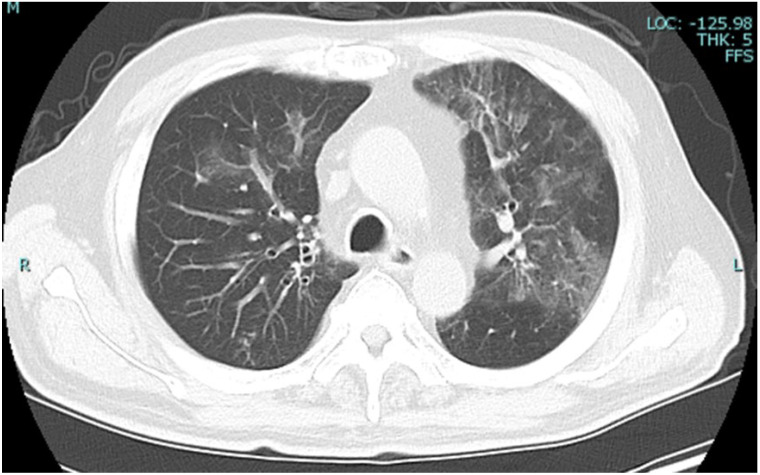
Chest CT on admission. Chest CT revealing non−segmental ground−glass opacities, predominantly in the upper lobes bilaterally. CT, computed tomography.

Chest radiography (Figure 1) revealed a cardiothoracic ratio of 55%, sharp right and dull left costophrenic angles, and diffuse ground-glass opacities in the right lung field. Chest computed tomography (CT) (Figure 2) revealed non-segmental ground-glass opacities, predominantly in the bilateral upper lobes.

### 2.2. Diagnosis and Differential Diagnosis

Given the ground-glass opacities and positive SARS-CoV-2 antigen test, recurrent COVID-19 pneumonia was considered the most likely diagnosis. Other viral pneumonias and an exacerbation of organizing pneumonia were ruled out based on blood and sputum culture results as well as viral antibody testing.

### 2.3. Treatment

Treatment was initiated with remdesivir (200 mg on day one, then 100 mg/day for 5 days), dexamethasone (6 mg/day for 10 days), and oxygen at 2 L/min via a nasal cannula. On hospital day 8, the SARS-CoV-2 antigen level had increased from 103 to 389 pg/mL. Lymphocyte subset analysis revealed profound B-cell depletion and CD4-+ T-cell reduction, with CD3 (80.4% [standard value: 72.3 ± 8.3%], absolute 88/µL), CD4 (18.0% [41.2 ± 6.7%], absolute 20/µL), CD8 (67.1% [22.0 ± 4.4%], absolute 73/µL), CD20 (0.1% [7.6 ± 4.7%], absolute 0.1/µL), and CD16 (13.0% [11.1 ± 3.3%], absolute 14/µL) expression. Serum Ig levels remained low (IgG, 589 mg/dL; IgA, 70 mg/dL; and IgM, 7 mg/dL). A 5000 mg dose of intravenous immunoglobulin (IVIG) was administered on days 12, 19, and 27, with each administration targeting an IgG level of ≥1000 mg/dL.

After the first IVIG administration on day 12 of the hospitalization, the SARS-CoV-2 antigen level did not decrease; instead, it rose to 3719 pg/mL by day 16, and the patient’s oxygen requirement remained unchanged at 2 L/min. The CRP level also increased from 1.8 to 4.1 mg/dL. Nevertheless, no improvement was observed in clinical symptoms (e.g., fever, cough, or sputum production) or in CRP levels. A second IVIG dose was administered on day 19, but IVIG alone did not lead to improvement in clinical symptoms or findings.

Therefore, from days 20 to 24, remdesivir (200 mg on day 1, followed by 100 mg/day) was continued, and ensitrelvir (375 mg on day 1, followed by 125 mg/day for 5 days) was added. The previously persistent symptoms—including fever, cough, and sputum production—showed marked clinical improvement.

### 2.4. Outcome and Follow-Up

**Figure 3:**
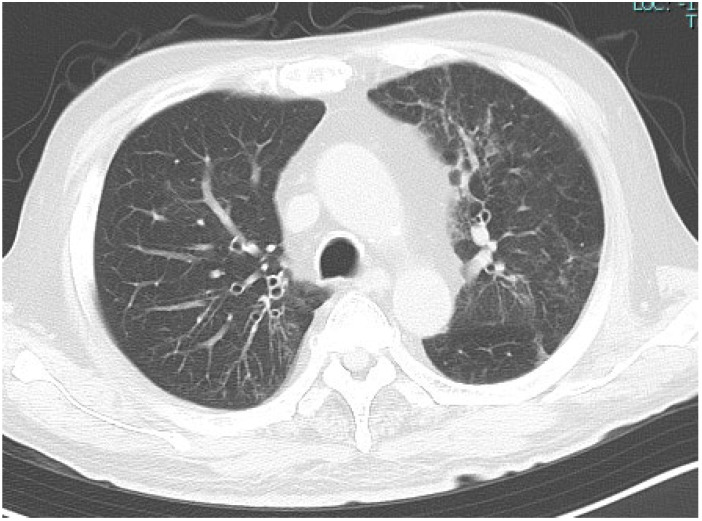
Chest CT on day 28. Chest CT showing improvement in the pneumonia findings. CT, computed tomography.

By day 21, oxygen supplementation was no longer required, and the patient maintained SpO_2_ of 95–97% on room air. By day 26, the SARS-CoV-2 antigen level had decreased from 3719 to 66.71 pg/mL, and the CRP level had improved to below 1 mg/dL. Chest CT on day 28 demonstrated significant improvement of pneumonia (Figure 3), and CRP had normalized. On day 29, the SARS-CoV-2 antigen level fell below the detection limit (<1.00 pg/mL), and the patient was discharged after overall clinical improvement was confirmed.

**Figure 4:**
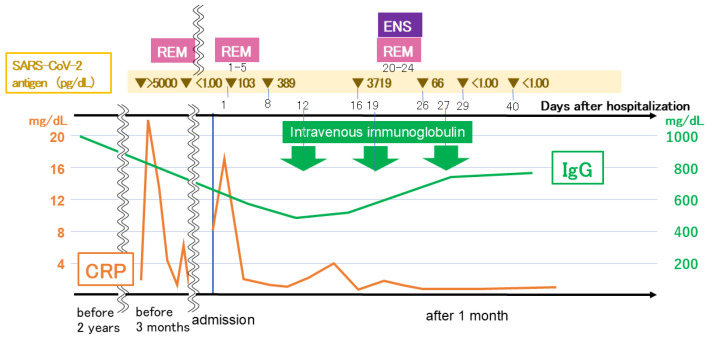
Clinical course. CRP, C-reactive protein; ENS, ensitrelvir; Ig, immunoglobulin; REM, remdesivir; SARS-CoV-2, severe acute respiratory syndrome coronavirus 2.

Based on the clinical course, the reduction in viral antigen levels and the improvement in symptoms and clinical findings were attributable to the antiviral agents, not to IVIG. The patient’s clinical course is shown in Figure 4.

After discharge, the patient was followed up regularly with IVIG when IgG levels fell below 600 mg/dL, in accordance with hematopoietic stem cell transplantation protocols [9]. One year after discharge, serum IgG levels remained stable at 600–700 mg/dL.

## 3. Discussion

We report a case in which ensitrelvir demonstrated marked efficacy in a patient exhibiting persistent CD20+ B-cell depletion and CD4+ T-cell reduction for several years following obinutuzumab therapy.

Ensitrelvir is a SARS-CoV-2 main protease (3CL pro) inhibitor with a mechanism similar to that of nirmatrelvir and ritonavir, with no major differences in antiviral activity reported [5]. Compared with remdesivir and molnupiravir, ensitrelvir can achieve better SARS-CoV-2 antigen clearance and more favorable viral shedding profiles [10]. Clinically, several case reports have described patients with malignant lymphoma who were refractory to remdesivir monotherapy but showed a rapid reduction in SARS-CoV-2 antigen levels and marked clinical improvement after receiving ensitrelvir [11,12,13,14].

Patients treated with anti-CD20 antibodies such as obinutuzumab often experience profound impairment of humoral immunity due to B-cell depletion, resulting in delayed B-cell recovery, impaired B-cell differentiation, hypogammaglobulinemia, and increased susceptibility to infection [8]. Although B-cell recovery typically occurs within several months to 1 year after the final dose, cases with persistent B-cell depletion lasting more than 1.5 years have been reported [15]. Obinutuzumab, a second-generation anti-CD20 antibody, induces more potent and sustained B-cell depletion than rituximab [16] and is associated with a higher incidence of hypogammaglobulinemia. In one study, patients treated with obinutuzumab had significantly higher rates of prolonged COVID-19 infection (38.9% vs. 2.9%) and more severe disease (33.3% vs. 4.7%) compared with those treated with rituximab [17].

Recent reports have begun to suggest the potential efficacy of ensitrelvir in immunocompromised states beyond B-cell-depleting therapy. In particular, several case reports have described immunocompromised patients, including those with hematologic malignancies, recipients of hematopoietic stem cell transplantation, and individuals on long-term corticosteroid therapy, in whom both cellular and humoral immunity may be impaired. These patients showed insufficient viral suppression with remdesivir alone but experienced a rapid decline in viral antigen levels and clinical improvement after the addition of ensitrelvir [11,12,13,14]. However, the available evidence is limited to case reports, and treatment responsiveness may vary according to the type of immunodeficiency, predominantly T- or B-cell dysfunction, or combined immunodeficiency. Therefore, the therapeutic role of ensitrelvir remains unestablished in specific immunosuppressed populations, and prospective studies stratified by the underlying type of immunodeficiency are needed.

In addition, our patient had previously received bendamustine, which is known to impair cellular immunity through T-cell depletion and CD4+ T-cell reduction [18,19]. Consistent with these findings, our patient exhibited marked CD4+ T-cell depletion. Thus, he was in a state of combined humoral and cellular immunodeficiency, likely resulting in an inadequate type I interferon response to SARS-CoV-2 and incomplete viral clearance, ultimately predisposing him to recurrent infection.

At present, no evidence indicates that ensitrelvir directly influences T-cell counts or functional T-cell responses. Its mechanism of action is limited to the inhibition of viral replication, and it is not known to modulate host immune reconstitution. In our patient, clinical improvement occurred despite persistent CD4+ T-cell depletion, suggesting that potent direct antiviral activity can lead to favorable outcomes even when adaptive immunity is severely impaired. Future studies evaluating immunologic parameters before and after ensitrelvir therapy in immunocompromised patients would help clarify whether indirect effects on immune function occur through reductions in viral burden.

Although more than 17 months had elapsed since his final dose of obinutuzumab, the patient continued to exhibit persistent B-cell depletion (<0.1%) and low IgG levels. This suggests the possibility of long-term immunosuppression following obinutuzumab treatment, reaffirming the need for years of monitoring for infection risk, even after treatment cessation.

SARS-CoV-2 antigen levels have been reported to correlate with disease severity and clinical outcomes, and serial antigen measurements may reflect changes in viral load and antiviral treatment efficacy [20]. At the initial presentation, 3 months prior to the current episode, the patient’s SARS-CoV-2 antigen level exceeded 5000 pg/mL and became negative after a course of remdesivir monotherapy. During the recurrence, the SARS-CoV-2 antigen level rose again, reaching a maximum of 3719 pg/mL. Although remdesivir monotherapy was administered once more, viral suppression remained insufficient. Following the approach reported by Jung et al. [12], ensitrelvir was added to remdesivir, resulting in a rapid decline in the antigen level from 3719 to 66 pg/mL, accompanied by marked improvement in inflammatory markers, fever, and oxygenation. These findings suggest that dual antiviral therapy with remdesivir and ensitrelvir enhances viral suppression in immunocompromised patients with impaired viral clearance. According to the guidelines [7], baricitinib and tocilizumab are recommended for moderate-to-severe pneumonia in which mechanical ventilation is anticipated. However, the severity of pneumonia in this patient did not meet these criteria. In addition, the patient had combined humoral and cellular immunodeficiency, and an insufficient type I interferon response to SARS-CoV-2 likely resulted in inadequate viral clearance, ultimately predisposing him to recurrent infection. In COVID-19, both baricitinib and tocilizumab are immunomodulatory agents intended to suppress excessive inflammatory responses (the cytokine storm), rather than acting directly on the virus itself [17]. For these reasons, we considered that baricitinib and tocilizumab were not indicated in this patient.

The rapid decline in SARS-CoV-2 antigen levels observed after ensitrelvir initiation may also have contributed to attenuation of the inflammatory response. Persistent viral replication in immunocompromised patients can sustain the production of proinflammatory cytokines such as IL-1β, TNF, and IFN-γ. Although ensitrelvir has no known direct immunomodulatory effects, a reduction in viral antigenic stimulation may cause a secondary decrease in cytokine production and inflammatory activity. This mechanism may partly explain the improvement in CRP levels and respiratory symptoms observed in our patient. Further research is needed to elucidate the relationship between viral antigen kinetics and cytokine dynamics during antiviral therapy.

Persistent SARS-CoV-2 infection in immunocompromised hosts is associated with ongoing viral replication and the emergence of novel variants [21]. Therefore, treatment strategies that achieve rapid viral load reduction are important not only for improving individual patient outcomes but also from a public health perspective. Ensitrelvir, with its potent antiviral activity and short treatment duration, may be a valuable therapeutic option for persistent or refractory COVID-19. As demonstrated in this case, combination therapy with ensitrelvir may be effective when responses to other antiviral agents are inadequate in immunocompromised patients.

## 4. Conclusions

This case involved a patient with follicular lymphoma who had undergone obinutuzumab plus bendamustine therapy and exhibited persistent hypogammaglobulinemia and profound B-cell depletion for more than 1 year after the final dose. The patient subsequently developed persistent and refractory COVID-19. Although management was challenging because of the patient’s immunocompromised state, the addition of ensitrelvir to remdesivir was associated with marked clinical efficacy. This case suggests that ensitrelvir may be a valuable therapeutic option for persistent or refractory COVID-19 in immunocompromised patients. Furthermore, clinicians should recognize that patients treated with obinutuzumab may remain immunosuppressed for several years, necessitating prolonged monitoring of their infection risk.

## Figures and Tables

**Table 1:** Laboratory data on admission.

Parameter	Recorded Value	Standard Value
White blood cell count	1520/µL	4500–7500/µL
Neutrophils	89.5% (absolute 1360/µL)	42–74%
Lymphocytes	7.2% (absolute 110/µL)	18–50%
Monocytes	2.6% (absolute 40/µL)	1–10%
Hemoglobin	13.5 g/dL	11.3–15.2 g/dL
Platelet count	16.8 × 10^4^/µL	13–35 × 10^4^/µL
Prothrombin time/International normalized ratio	1.03	0.80–1.20
Activated partial thromboplastin time	32.9 s	26.9–38.1 s
D-dimer	1.7 μg/mL	<1.0 μg/mL
C-reactive protein	7.98 mg/L	≤0.60 mg/dL
Total protein	5.7 g/dL	6.9–8.4 g/dL
Albumin	3.1 g/dL	3.9–5.1 g/dL
Total bilirubin	0.8 mg/dL	0.2–1.2 mg/dL
Aspartate aminotransferase	28 U/L	11–30 U/L
Alanine aminotransferase	32 U/L	4–30 U/L
Lactase dehydrogenase	423 U/L	109–216 U/L
Creatine kinase	25 U/L	40–150 U/L
Blood urea nitrogen	12.8 mg/dL	8–20 mg/dL
Creatinine	0.69 mg/dL	0.63–1.03 mg/dL
Sodium	142 mEq/L	136–148 mEq/L
Potassium	3.6 mEq/L	3.6–5.0 mEq/L
Chloride	98 mEq/L	98–108 mEq/L
Glucose	366 mg/dL	70–109 mg/dL
SARS-CoV-2 antigen	103.63 pg/dL	<1.0 pg/dL
Cytomegalovirus IgM	(−)	
Cytomegalovirus IgG	(+)	
Herpes Simplex virus IgM	(−)	
Herpes Simplex virus IgG	(−)	
Varicella–Zoster virus IgM	(−)	
Varicella–Zoster virus IgG	(+)	
Human T-cell Leukemia virus type 1/2 antibiotics	(−)	
Human immunodeficiency virus antibiotics	(−)	
Mycoplasma nucleic acid (pharyngeal), LAMP method	(−)	
Urinary Legionella antigen	(−)	
Sputum *Pneumocystis jirovecii* deoxyribonucleic acid	(−)	
Beta-D-glucan	<4.0	<4.0
IgG	589 mg/dL	861–1747 mg/dL
IgA	70 mg/dL	93–393 mg/dL
IgM	7 mg/dL	33–183 mg/dL

Ig, immunoglobulin; SARS-CoV-2, severe acute respiratory syndrome coronavirus 2; (+), postive; (−), negative.

## Data Availability

The original data presented in the study are included in the article, further inquiries can be directed to the corresponding author.

## Abbreviations

The following abbreviations are used in this manuscript:

COVID-19	coronavirus disease 2019
SARS-CoV-2	severe acute respiratory syndrome coronavirus 2
CT	computed tomography
CRP	C-reactive protein

## References

[1] World Health Organization Classification of Omicron (B.1.1.529): SARS-CoV-2 Variant of Concern. https://www.who.int/news/item/26-11-2021-classification-of-omicron-(b.1.1.529)-sars-cov-2-variant-of-concern.

[2] Beigel J.H., Tomashek K.M., Dodd L.E., Mehta A.K., Zingman B.S., Kalil A.C., Hohmann E., Chu H.Y., Luetkemeyer A., Kline S. (2020). Remdesivir for the Treatment of COVID-19-Final Report. N. Engl. J. Med..

[3] Jayk Bernal A., Gomes da Silva M.M., Musungaie D.B., Kovalchuk E., Gonzalez A., Delos Reyes V., Martín-Quirós A., Caraco Y., Williams-Diaz A., Brown M.L. (2022). Molnupiravir for Oral Treatment of COVID-19 in Nonhospitalized Patients. N. Engl. J. Med..

[4] Hammond J., Leister-Tebbe H., Gardner A., Abreu P., Bao W., Wisemandle W., Baniecki M., Hendrick V.M., Damle B., Simón-Campos A. (2022). Oral Nirmatrelvir for High-Risk, Nonhospitalized Adults with COVID-19. N. Engl. J. Med..

[5] Kuroda T., Nobori H., Fukao K., Baba K., Matsumoto K., Yoshida S., Tanaka Y., Watari R., Oka R., Kasai Y. (2023). Efficacy comparison of 3CL protease inhibitors ensitrelvir and nirmatrelvir against SARS-CoV-2 in vitro and in vivo. J. Antimicrob. Chemother..

[6] Yotsuyanagi H., Ohmagari N., Doi Y., Yamato M., Bac N.H., Cha B.K., Imamura T., Sonoyama T., Ichihashi G., Sanaki T. (2024). Efficacy and Safety of 5-Day Oral Ensitrelvir for Patients with Mild to Moderate COVID-19: The SCORPIO-SR Randomized Clinical Trial. JAMA Netw. Open.

[7] Clinical Management of Patients with COVID-19: A Guide for Front-Line Healthcare Workers (Version 10.1). [2023 Health and Labour Policy Promotion Survey Grant, Emerging and Re-Emerging Infectious Diseases and Vaccination Policy Promotion Research Project, Research Regarding Clinical Measures in Preparation for Occurrence of Category I Infectious Disease Patients]. https://www.mhlw.go.jp/content/001248424.pdf.

[8] Blincoe A., Labrosse R., Abraham R.S. (2022). Acquired B-cell deficiency secondary to B-cell-depleting therapies. J. Immunol. Methods.

[9] Karakulska-Prystupiuk E., Dwilewicz-Trojaczek J., Drozd-Sokołowska J., Kmin E., Chlebus M., Szczypińska K., Boguradzki P., Tomaszewska A., Mądry K., Biliński J. (2021). Prevalence of hypogammaglobulinemia and its management with subcutaneous immunoglobulin supplementation in patients after allogeneic hematopoietic stem cell transplantation-a single-center analysis. Ann. Hematol..

[10] Yoshida R., Sasaki T., Ohsaki Y. (2024). Real-World Efficacy of Ensitrelvir in Hospitalized Patients with COVID-19 in Japan: A Retrospective Observational Study. Cureus.

[11] Seki M., Mitsutake K., Shimizu A., Honda D., Ishigami K., Yamanaka M., Kuwata Y., Ueda G., Enami K. (2023). Ensitrelvir improved SARS-CoV-2 viral titers of COVID-19 patients refractory to remdesivir. World J. Clin. Med. Res..

[12] Jung S., Yagi Y., Fukushima K., Nishikawa Y., Tanaka M., Kobayashi T., Yajima K., Ajisawa A., Imamura A. (2023). Successful dual antiviral therapy with remdesivir and ensitrelvir in a case of prolonged COVID-19 following B-cell depleting immunotherapy for malignant lymphoma. IDCases.

[13] Sakamaki I., Negoro E., Iwasaki H., Yamauchi T. (2024). Ensitrelvir eradicates persistent SARS-CoV-2 infection in a follicular lymphoma patient treated with anti-CD20 antibodies. J. Infect. Chemother..

[14] Furuya C., Yasuda H., Hiki M., Shirane S., Yamana T., Uchimura A., Inano T., Takaku T., Hamano Y., Ando M. (2024). Case report: Ensitrelvir for treatment of persistent COVID-19 in lymphoma patients: A report of two cases. Front. Immunol..

[15] Furie R.A., Aroca G., Cascino M.D., Garg J.P., Rovin B.H., Alvarez A., Fragoso-Loyo H., Zuta-Santillan E., Schindler T., Brunetta P. (2022). B-cell depletion with obinutuzumab for the treatment of proliferative lupus nephritis: A randomised, double-blind, placebo-controlled trial. Ann. Rheum. Dis..

[16] Goede V., Klein C., Stilgenbauer S. (2015). Obinutuzumab (GA101) for the treatment of chronic lymphocytic leukemia and other B-cell non-hodgkin’s lymphomas: A glycoengineered type II CD20 antibody. Oncol. Res. Treat..

[17] Shu W., Yang Q., Le J., Cai Q., Dai H., Luo L., Tong J., Song Y., Chen B., Chen D. (2024). Outcomes of COVID-19 in patients with obinutuzumab compared with patients with rituximab: A retrospective cohort study. Virol. J..

[18] Saito H., Maruyama D., Maeshima A.M., Makita S., Kitahara H., Miyamoto K., Fukuhara S., Munakata W., Suzuki T., Kobayashi Y. (2015). Prolonged lymphocytopenia after bendamustine therapy in patients with relapsed or refractory indolent B-cell and mantle cell lymphoma. Blood Cancer J..

[19] Gafter-Gvili A., Polliack A. (2016). Bendamustine associated immune suppression and infections during therapy of hematological malignancies. Leuk. Lymphoma.

[20] Favresse J., Bayart J.L., David C., Gillot C., Wieërs G., Roussel G., Sondag G., Elsen M., Eucher C., Dogné J.M. (2022). Serum SARS-CoV-2 Antigens for the Determination of COVID-19 Severity. Viruses.

[21] Hettle D., Hutchings S., Muir P., Moran E., COVID-19 Genomics UK (COG-UK) consortium (2022). Persistent SARS-CoV-2 infection in immunocompromised patients facilitates rapid viral evolution: Retrospective cohort study and literature review. Clin. Infect. Pract..

